# Disrupting polycystin-2 EF hand Ca^2+^ affinity does not alter channel function or contribute to polycystic kidney disease

**DOI:** 10.1242/jcs.255562

**Published:** 2020-12-24

**Authors:** Thuy N. Vien, Leo C. T. Ng, Jessica M. Smith, Ke Dong, Matteus Krappitz, Vladimir G. Gainullin, Sorin Fedeles, Peter C. Harris, Stefan Somlo, Paul G. DeCaen

**Affiliations:** 1 Department of Pharmacology, Feinberg School of Medicine, Northwestern University, Chicago, IL 60611, USA; 2 Division of Nephrology and Hypertension, Mayo Clinic, Rochester, MN 55905, USA; 3 Departments of Internal Medicine and Genetics, Yale University School of Medicine, New Haven, CT 06510, USA

**Keywords:** ADPKD, Biophysics, Ca^2+^ regulation, Ion channels, Polycystin, Primary cilia

## Abstract

Approximately 15% of autosomal dominant polycystic kidney disease (ADPKD) is caused by variants in *PKD2*. *PKD2* encodes polycystin-2, which forms an ion channel in primary cilia and endoplasmic reticulum (ER) membranes of renal collecting duct cells. Elevated internal Ca^2+^ modulates polycystin-2 voltage-dependent gating and subsequent desensitization – two biophysical regulatory mechanisms that control its function at physiological membrane potentials. Here, we refute the hypothesis that Ca^2+^ occupancy of the polycystin-2 intracellular EF hand is responsible for these forms of channel regulation, and, if disrupted, results in ADPKD. We identify and introduce mutations that attenuate Ca^2+^-EF hand affinity but find channel function is unaltered in the primary cilia and ER membranes. We generated two new mouse strains that harbor distinct mutations that abolish Ca^2+^-EF hand association but do not result in a PKD phenotype. Our findings suggest that additional Ca^2+^-binding sites within polycystin-2 or Ca^2+^-dependent modifiers are responsible for regulating channel activity.

## Abstract (precis)

**Summary:** A refutation of a common hypothesis regarding Ca^2+^ control mechanisms of polycystin-2 – a primary cilia ion channel, the dysregulation of which causes autosomal dominant polycystic kidney disease.

## INTRODUCTION

*PKD2* encodes for polycystin-2, a member of the polycystin subfamily of transient receptor potential ion channels (TRPP) (Venkatachalam and Montell, 2007). Previous work has established that polycystins form Ca^2+^-conducting channels in the primary cilia membrane of disparate tissues (DeCaen et al., 2013; Kleene and Kleene, 2012, 2017; Liu et al., 2018). Primary cilia are solitary projections that extend ∼5 μm from the apical side of cells. They are privileged cellular organelles comprising fewer than 500 unique protein components, and are found in all organ systems, including cells of the kidney nephron (Ostrowski et al., 2002; Pazour et al., 2005). Primary cilia house specific receptors and downstream effectors, which are capable of modulating morphogenic gene transcription responsible for left-right axis determination in developing vertebrate embryos (Braun and Hildebrandt, 2017; Mizuno et al., 2020; Nachury and Mick, 2019). Dysregulation of cilia- and centrosome-localized proteins results in ciliopathies – conditions that often affect specific organ systems but commonly share cystic kidney diseases as a comorbidity (Hildebrandt et al., 2011; Hildebrandt and Zhou, 2007). Variants in human *PKD2* account for ∼15% of cases of autosomal dominant polycystic kidney disease (ADPKD) (Grantham, 2001; Mochizuki et al., 1996), a common ciliopathy that is characterized by progressive cyst development, which ultimately causes renal failure. ADPKD is proposed to be a recessive disease at the cellular level in which individuals inherit one variant polycystin allele and develop cysts after acquiring a second somatic mutation in the remaining allele (Koptides et al., 1999; Pei, 2001). Mouse models of attenuating *Pkd2* expression by conditional genetic repression faithfully recapitulate the polycystic kidney phenotype (Happé and Peters, 2014; Ma et al., 2013; Wilson, 2008; Wu et al., 1998), whereas complete genetic deletion of *Pkd2* causes embryonic lethality and kidney cyst development *in utero* (Menezes and Germino, 2013; Wu et al., 2000). These studies in mice suggest that most ADPKD-causing variants are loss of function, but our understanding of their mechanistic impact remains largely undetermined owing to their subcellular localization.

The primary cilium is electrochemically discrete from the cytosol, having its own depolarized membrane potential and resting concentration of Ca^2+^ (DeCaen et al., 2013; Delling et al., 2013). Analyzing polycystin-2 in primary cilia has opened opportunities to study its function under endogenous and heterologous expression conditions (Kleene and Kleene, 2017; Liu et al., 2018). Most recently, this methodology was used to determine that several disease-causing variants found in the human ADPKD population alter polycystin-2 gating without affecting its ciliary localization and tetramerization (Vien et al., 2020). Polycystin-2 forms a voltage-gated channel that opens at positive membrane potentials. As the cilia resting membrane potential is ∼−17 mV, most channels are closed until internal Ca^2+^ is elevated to micromolar concentrations (Delling et al., 2013). Once elevated, the voltage required to open polycystin-2 becomes hypopolarized and channel closure becomes incomplete at negative membrane potentials (Kleene and Kleene, 2017; Liu et al., 2018). In this article, we use the term ‘Ca^2+^-dependent modulation’ (CDM) to describe this form of polycystin-2 gating regulation. Importantly, this mechanism gives polycystin-2 gating its physiological relevance, allowing Ca^2+^ and monovalent ions to flow into the primary cilium at its resting membrane potential. Subsequently, prolonged and elevated intraciliary Ca^2+^ causes polycystin-2 channels to enter an irreversible non-conducting state, through a process we have termed ‘Ca^2+^-dependent desensitization’ (CDD). This form of polycystin regulation was postulated to protect the cell from Ca^2+^ overload, by turning off the flow of Ca^2+^ at the source within the ciliary compartment (DeCaen et al., 2013, 2016). Although CDD has been attributed to Ca^2+^ occupancy of the related polycystin-20-like 1 pore, the structural determinants responsible for CDD and CDM of polycystin-2 are unknown.

Besides primary cilia, polycystin-2 activity has been reported in the membranes of intracellular organelles (Koulen et al., 2002; Kuo et al., 2019). Polycystin-2 functionally mimics the Ca^2+^ response from bona fide endoplasmic reticulum (ER) ion channels, so its contribution is difficult to distinguish from inositol trisphosphate (IP3R)- and ryanodine receptor channel-mediated effects (Koulen et al., 2002; Qian et al., 2003; Vassilev et al., 2001). Measuring polycystin-2 in bilayers from reconstituted ER membranes is challenging, in part because it directly associates with IP3R (Santoso et al., 2011). Results from reconstitution and cytosolic Ca^2+^ assays have demonstrated that ER-localized polycystin-2 channels are responsive to intracellular Ca^2+^, and that mutations that remove the entire C-terminal domain (CTD), or alter the EF hand, abolish the entire function of the channel (Ćelić et al., 2012; Koulen et al., 2002). Partly based on this work, it was proposed that Ca^2+^ occupancy of its C-terminal EF hand is requisite for polycystin-2 channel function and Ca^2+^-dependent activation (Ćelić et al., 2012; Koulen et al., 2002; Petri et al., 2010; Yang and Ehrlich, 2016). However, these results have neither been validated from the ciliary pool of polycystin-2, nor have they provided a mechanistic description of the regulation of channel gating by Ca^2+^. In this study, we determined which EF hand vertices are most important for Ca^2+^ affinity. We found that disrupting Ca^2+^ occupancy of the EF hand did not alter CDM or CDD of the mouse and human orthologs of polycystin-2 in the primary cilia. We characterized the kidney phenotype from two new mice that express unique mutations that disrupt Ca^2+^-EF hand association but fail to develop polycystic kidney disease. We measured Gα_q_-mediated Ca^2+^ release from primary collecting duct cells isolated from wild-type and mutant mice to test ER-localized populations of polycystin-2, but found no difference. Our results suggest that Ca^2+^-dependent biophysical regulation of polycystin-2 involves other sites and/or effector proteins that control channel opening and desensitization. Our findings demonstrate that disruption of Ca^2+^-EF hand affinity does not lead to impaired *in vitro* or *in vivo* function of polycystin-2, which suggests that ADPKD-causing truncating variants found in the CTD likely affect other motifs that have a greater impact on channel regulation.

## RESULTS

**Fig. 1.**
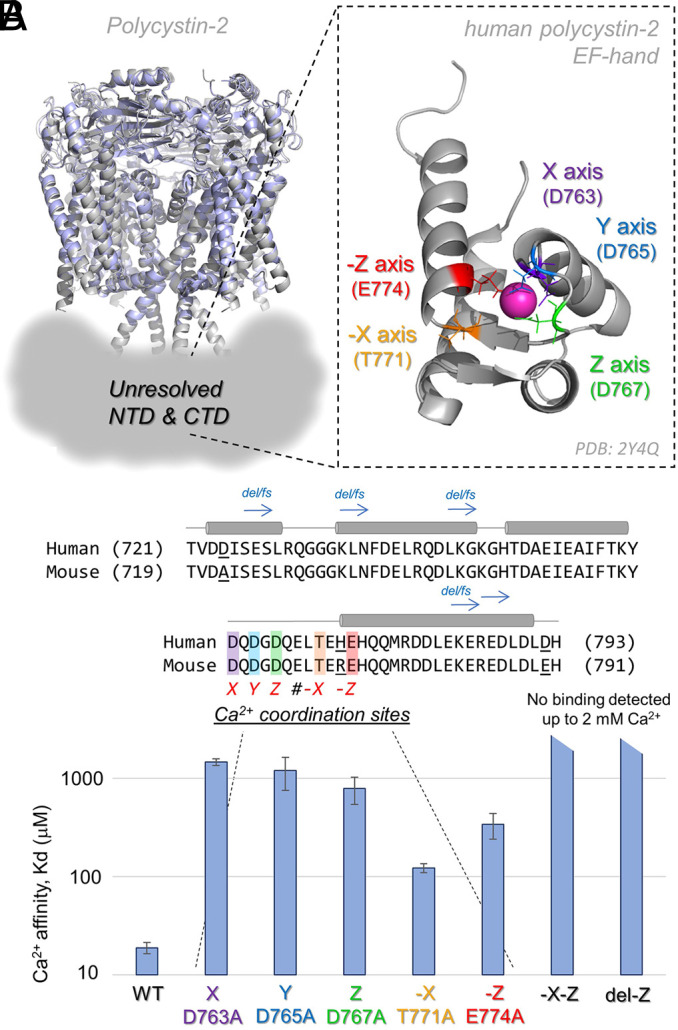
**Disruption of Ca^2^ affinity for the polycystin-2 EF hand by single alanine substitutions and mouse model deletion and double mutations.** (A) Structural alignment of two polycystin-2 channel structures solved using cryo-EM (PDBs: 5MKE in gray and 5T4D in light blue) (Shen et al., 2016; Wilkes et al., 2017). The hypothetical location of the unresolved internal CTD, which contains coiled-coil and EF hand motifs, is represented by a gray density. Inset, the human polycystin-2 EF hand structure solved by solution NMR (PDB: 2Y4Q, residues 721-793) with the Ca^2+^-coordinating vertices highlighted (Allen et al., 2014). (B) Top, an alignment of the human and mouse polycystin-2 EF hands. The locations of ADPKD-associated truncating frame shift variants are indicated by blue arrows. The location of the mouse model deletion (del-Z) and double alanine substitution (-X-Z) mutations used in this study are indicated. Bottom: calorimetry results measuring the differences in Ca^2+^-EF hand affinity when the coordinating vertices are substituted for alanine in the human CTD fragment (704-797). Average calculated Ca^2+^ affinity (*K*_d_) from six replicates per peptide are graphed (data are mean±s.d.). Angled bar graph caps indicated that no affinity was detected from mutant EF hand fragments when tested up to 2 mM Ca^2+^.

Two groups have independently reported that human and mouse orthologs of polycystin-2 are activated by internal Ca^2+^ using direct cilia electrophysiology (Kleene and Kleene, 2017; Liu et al., 2018). Polycystin-2 channels from both species are highly conserved and contain a C-terminal Ca^2+^-binding EF hand domain – a structural motif that is proposed to confer Ca^2+^ sensitivity in these channels (Ćelić et al., 2012; Mochizuki et al., 1996; Yang and Ehrlich, 2016). Although this motif has escaped structural determination in previously published cryogenic electron microscopy (cryo-EM) polycystin-2 structures, isolated EF hand(s) from human and sea urchin orthologs have been determined using crystallographic and nuclear magnetic resonance (NMR) methods (Allen et al., 2014; Petri et al., 2010; Yu et al., 2009). The EF hand of human polycystin-2 has five conserved vertices (X, Y, Z, -X and -Z) that coordinate the Ca^2+^ ion with four side chain carboxylate and one backbone carbonyl interactions (Fig. 1A,B). We isolated a CTD peptide of human polycystin-2 (I704-P797) that contains the EF hand, and measured changes in heat capacity upon Ca^2+^ addition using isothermal titration calorimetry (Fig. S1A). We determined that the affinity of Ca^2+^ for the wild-type human CTD EF hand was 19±5 µM – a finding which approximates the value previously reported (*K*_d_=22 µM) for this peptide (Yang et al., 2015). Importantly, the polycystin-2 EF hand did not have affinity for other physiologically relevant divalent ions, Mg^2+^ and Zn^2+^ (Fig. S1B). We then individually mutated the EF hand vertices to alanine to determine which positions contribute to Ca^2+^ binding. We found that all five vertices are involved in Ca^2+^ affinity for the EF hand, as all alanine substitutions increased *K*_d_ 6–68× (Fig. 1B; Table S1). Based on these results, if Ca^2+^ occupancy of the EF hand regulates polycystin-2 function, disrupting its affinity for this motif should translate into measurable changes in the Ca^2+^-dependent properties of the channel.

**Fig. 2.**
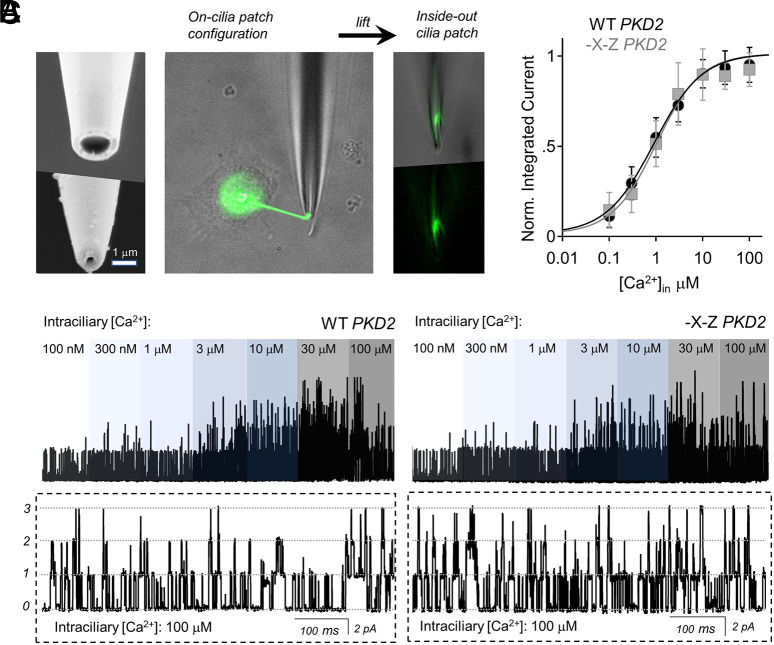
**Intraciliary Ca^2+^ activates primary cilia currents conducted by** **wild-type** **and -X-Z polycystin-2 channels with similar potency.** (A) Establishing high resistance seals with primary cilia in the inside-out configuration. Left: scanning electron micrograph images of a cilia patch electrode before (top) and after (bottom) fire polishing. Middle: images of a voltage-clamped primary cilia from an HEK cell expressing *PKD2-GFP*. Green fluorescence signal illuminates the cilia and intracellular compartments. Right: the tip of the cilia is lifted and torn from the cell to establish the inside-out configuration, in which intraciliary Ca^2+^ was exchanged in the perfusate. (B) Exemplar wild-type (WT) (left) or -X-Z (right) human polycystin-2 single channel currents recorded after exposure to increasing intraciliary Ca^2+^ while voltage clamped at 50 mV. Bottom: stochastic open channel events from the expanded time scale of the 100 µM Ca^2+^ condition. (C) The relationship of intraciliary Ca^2+^ and normalized integrated current from wild-type and -X-Z channels (*n*=8 cilia; data are mean±s.d.).

**Fig. 3.**
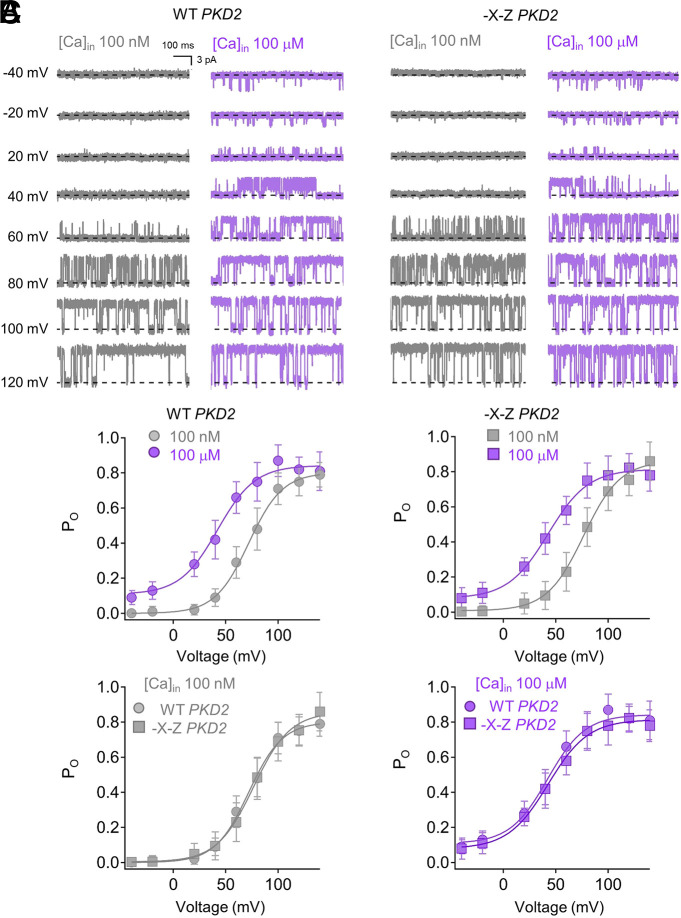
**Ca^2+^-dependent modulation of human polycystin-2 is unchanged after abolishing Ca^2+^-EF hand affinity using the -X-Z mutations.** (A) Exemplar open channel events captured from inside-out patches with one channel in the cilia membrane. (B,C) Average channel open probability (P_o_) plotted as a function of voltage in the presence of 100 nM and 100 µM intracellular Ca^2+^. The data were fitted using the Boltzmann equation to estimate the half maximal voltage response (*V*_1/2_). Data are mean±s.d. (*n*=8 cilia recordings from each channel type).

To test whether Ca^2+^ occupancy of the EF hand is responsible for CDM and CDD of human polycystin-2, we generated double alanine mutations at the -X (T771A) and -Z vertices (E774A), which abolished Ca^2+^ affinity for the CTD fragment (Fig. 1B). We then expressed the -X-Z double mutation in PKD2-GFP in a *PKD2*^null^ HEK cell line, so that exogenous channels could be assayed without the contribution of endogenous *PKD2* alleles, as described previously (Vien et al., 2020). The stably expressed human wild-type and -X-Z channels trafficked to the primary cilia, and glass electrodes with submicron apertures were fabricated to form high resistance electrical seals with the GFP-illuminated cilia membranes (Fig. 2A; Movie 1). As described previously, we established inside-out cilia patch configurations so that Ca^2+^ within the cilia (intraciliary) can be adjusted by the superfusate (Fig. 2A,B) (Liu et al., 2018). We observed that the number of single channel events increased when intraciliary Ca^2+^ was elevated from 100 nM to 100 µM for both wild-type and -X-Z channels (Fig. 2B,C). However, no difference in Ca^2+^ potency (EC_50_) for opening between wild-type and mutant channels was observed after fitting the integrated single channel activity to the Hill equation (Fig. 2C; Table S2). Previous work has established that CDM is a product of a hypopolarizing-shift in the voltage dependence of polycystin-2, which increases channel open probability (P_o_) at negative membrane potentials (Kleene and Kleene, 2017; Liu et al., 2018). To determine whether this mechanism was altered by the -X-Z mutation, we compared voltage-dependent channel opening at low (100 nM) and high (100 µM) intraciliary Ca^2+^ concentrations (Fig. 3A). These experiments were performed with only one channel present in the membrane so that stochastic effects on single channel states could be determined. The shift in the voltage-dependence of half maximal open probability (▵*V*_1/2_) from low to high Ca^2+^ was −47 mV for wild-type channels, and was not different from the shift observed in -X-Z channels (Fig. 3B,C; Table S2). As reported previously, we observed that polycystin-2 enters an irreversible desensitized state (CDD) after prolonged exposure to high internal Ca^2+^ (Liu et al., 2018). To test the EF hand role in this form of channel regulation, we conducted whole-cilia recordings using 30 µM intraciliary free Ca^2+^ and plotted the time course of current desensitization (Fig. S2A,C). The time course of CDD was not different between wild-type and -X-Z mutant channels, as the magnitude of cilia current was completely abolished for both channels after 3.5 min of recording. Taken together, these results demonstrate that abolishing Ca^2+^ occupancy of the human polycystin-2 EF hand does not alter CDM and CDD forms of channel regulation.

**Fig. 4.**
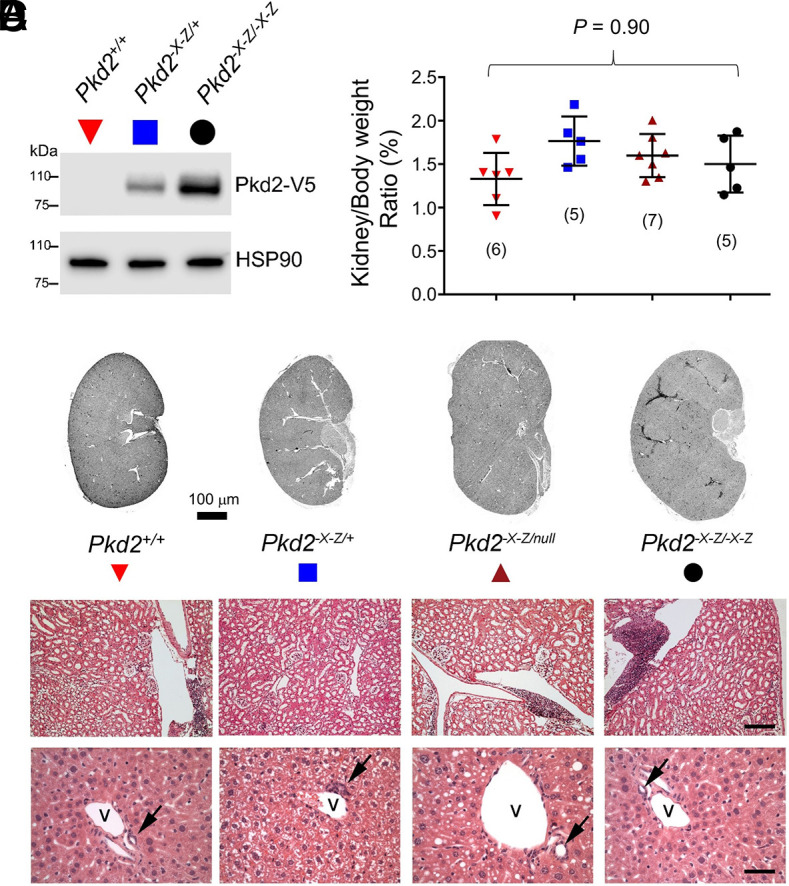
***Pkd2^-X-Z/-X-Z^* mice do not develop polycystic kidney or polycystic liver disease.** (A) Immunoblotting with anti-V5 antibody showing the absence of epitope-tagged Pkd2 signal in wild-type kidney (red inverted triangle), and dose-dependent expression of Pkd2-V5 in *Pkd2^-X-Z/+^* (blue square) and *Pkd2^-X-Z/-X-Z^* (black circle) kidney lysates. (B) Kidney-to-body weight ratio for 9-month-old wild-type (red inverted triangle), *Pkd2^-X-Z/+^* (blue square), *Pkd2^-X-Z/-X-Z^* (brown triangle) and *Pkd2^-X-Z/^*^null^ (black circle) mice. The *P*-value resulting from a one-way ANOVA statistical analysis is shown above the plot. The number of animals per genotype used are indicated within the parentheses. (C,D) Representative kidney scans (C) and hematoxylin and eosin (H&E) staining (D) of kidney sections (D, top row) and periportal liver sections (D, bottom row) from 9-month-old mice with the indicated (color coded) genotypes. Arrows indicate normal bile ducts; v, periportal vein. Scale bars: 100 µm (D, top row); 25 µm (D, bottom row).

Previous work disrupting polycystin-2 function by either allelic ablation or truncation of *Pkd2* resulted in embryonic lethality for homozygous mice (Wu et al., 1998, 2000). Conditional and kidney-specific ablation of *Pkd2* results in penetrant and reproducible polycystic kidney phenotype in mice (Ma et al., 2013). As the -X-Z mutation had no impact on polycystin-2 function, we hypothesized that homozygous animals expressing analogous mutations would likely survive, and the allelic impact of abolishing Ca^2+^ binding on cystogenesis in the kidney could be assessed *in vivo*. Furthermore, as the most sensitive bioassay for loss of polycystin function is cyst formation, we sought to determine whether the normal channel function without EF hand Ca^2+^ affinity is consistent with normal *in vivo* function by generating the equivalent -X-Z knock-in mouse model. To make the *Pkd2^-X-Z^* mouse, we employed the CRISPR/Cas9 method to replace the -X (T769A) and -Z (E772A) vertices, and simultaneously insert a V5 epitope tag immediately before the termination codon in murine *Pkd2* (Tran et al., 2019). We confirmed expression of the mutated epitope-tagged protein in the kidney lysates by immunoblot analysis with an anti-V5 antibody (Fig. 4A). The animals were viable without developing either kidney or liver cysts up to 18 months (data not shown). Mice were systematically examined at 9 months of age, and exhibited normal kidney weight and liver weight as a fraction of body weight, and normal histological appearance without evidence of kidney tubular bile duct dilation or cyst formation (Fig. 4B-D). *Pkd2^+/−^* mice were crossed with *Pkd2^-X-Z/-X-Z^* animals to generate *Pkd2^-X-Z/^*^Null^ mice to examine whether the reduced dosage could elicit a phenotype. These mice also did not display kidney or liver cysts at 9 months of age. The aggregate biophysical and *in vivo* results demonstrate that abolishing Ca^2+^-EF hand affinity using -X-Z vertices neutralizing mutations neither alters polycystin-2 ion channel function, nor engenders sufficient loss of function to cause polycystic kidney disease in mice.

**Fig. 5.**
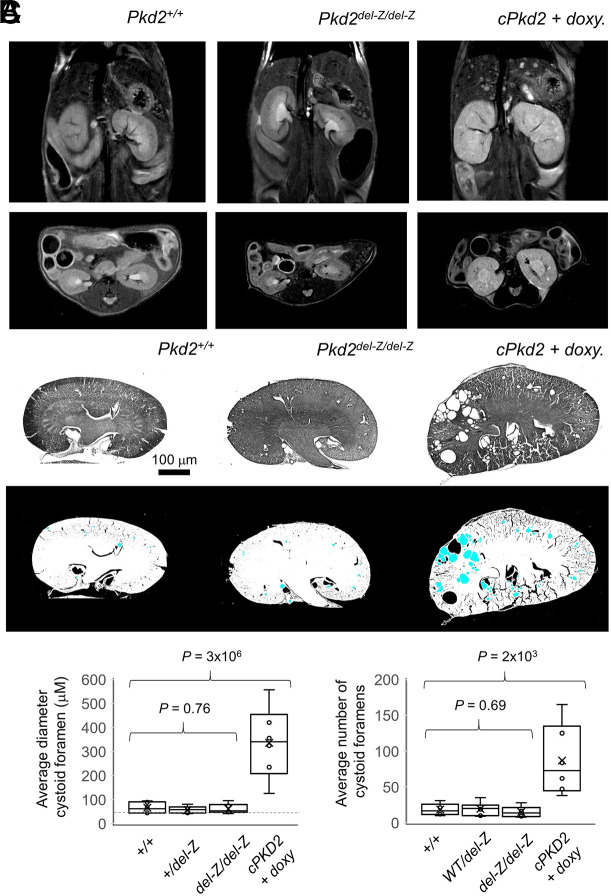
***Pkd2^del-Z/del-Z^* mice do not develop polycystic kidney disease.** (A) Exemplar MRI images of the frontal (top) and transverse (bottom) plane of the thoracic cavity of 12-month-old mice expressing the indicated *Pkd2* genotypes. Kidney and liver cysts are visible in both planes obtained from conditional *cPkd2* knockout animals (*Pax8^rtTA^; TetO-cre; Pkd2^fl/fl^*) 6 months after induction with doxycycline, as described previously (Liu et al., 2018; Ma et al., 2013). However, no obvious cysts were detected from mice expressing constitutive *Pkd2^del-Z^* alleles. (B) Top, exemplar black and white images of H&E-stained medial kidney sections. Bottom, FIJI analysis results were used to identify cystoid foramen (blue) after reverse-negative processing of the above images (Schindelin et al., 2012). (C) Box (s.e.m.) and whisker (s.d.) plots of the average diameter (left) and number (right) of foramen from kidney sections from 12-month-old mice (*n*=6 mice per genotype). Results from each individual mouse are indicated by open circles and the average for the geneotype is indicated by an x. *P*-values resulting from a one-way ANOVA statistical analysis are shown above the plots.

This result excludes the hypothesis that Ca^2+^ occupancy of the EF hand is necessary for polycystin-2 function, and when disrupted, contributes to polycystic kidney disease (PKD). Thus, we challenged the robustness of this interpretation by generating a second mouse strain (*Pkd2^del-Z^*), in which the EF hand -Z vertex (E772), along with the preceding arginine (R771) were genetically deleted. Using isothermal titration calorimetry measurements, we confirmed that the del-Z mutation abolished Ca^2+^ occupancy of the EF hand (Fig. 1B; Fig. S1B). *Pkd2^+/del-Z^*, *Pkd2^del-Z/del-Z^* and *Pkd2^del-Z/-^* mice were viable to at least 12 months of age, and the kidney morphology of these mice was periodically imaged using MRI (Fig. 5A). As a positive control, we imaged the development of PKD cysts from *cPkd2* mice, in which the expression of *Pkd2* was attenuated by doxycycline induction after 6 months of life (Ma et al., 2013). Development of PKD-type kidney cysts was apparent in all (10/10) of the induced *cPkd2* mice after one year, whereas none of the *Pkd2^+/+^* (0/18), *Pkd2^+/del-Z^* (0/19) and *Pkd2^del-Z/del-Z^* (0/18) animals developed PKD-type cysts. In our preliminary observations, a minority of the *Pkd2^del-Z/del-Z^* (4/35) mice developed bilateral cystic disease (non-PKD-type), but because of the infrequency of this phenotype and because these cysts were not observed after outbreeding, we conclude that this bilateral cyst phenotype is not likely caused by the del-Z mutation. Owing to the limited resolution of MRI, we conducted histology experiments on sectioned kidneys from six mice from each genotype. Consistent with the MRI results, we did not observe any obvious cysts in the kidney sections from the other mice showing homozygous expression for *Pkd2^del-Z^* alleles (Fig. 5B). To quantify this observation, we conducted image analysis of the average number and size of cystoid foramen in the sections (Fig. 5C). All *cPkd2* mice had significantly more and larger cystoids, whereas the sections isolated from mice expressing one or two copies of the *Pkd2^del-Z^* allele were not different from wild-type animals. Our results, generated from two mouse strains expressing unique mutations, demonstrate that abolishing Ca^2+^ affinity for the EF hand does not produce polycystic kidney disease *in vivo*.

**Fig. 6.**
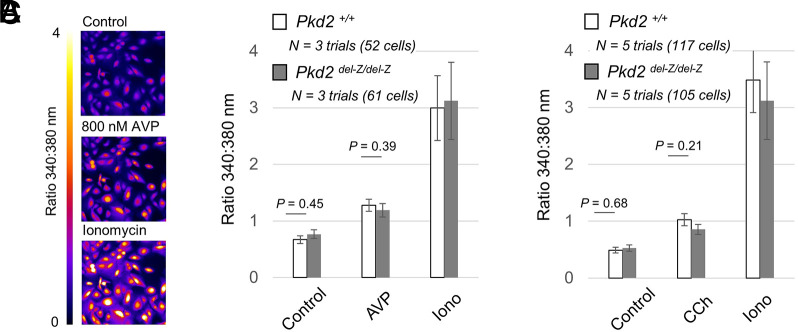
**Gα_q_-mediated Ca^2+^ release from ER stores are the same in collecting duct cells isolated from wild-type and *Pkd2^del-Z/del-Z^* mice.** (A) An exemplar Ca^2+^-dependent Fura-2 emission ratio (340:380 nm) from primary cultures of pIMCD cells isolated from mice *Pkd2^+/+^* mice. (B,C) Average cytosolic Fura-2 emission ratios from pIMCD cells isolated from *Pkd2^+/+^* and Pkd2^del-Z/del-Z^ mice before and after Gα_q_-mediated Ca^2+^ release using 800 nM arginine vasopressin (AVP) (B) or 50 µM carbachol (CCh) (C). The number of trials (*N*) and cells tested are listed per test group. Data are mean±s.d. *P*-values are indicated above the bar graphs (Student's *t*-test).

In our previous experiments, we established that the disruption of Ca^2+^-EF hand affinity does not alter the biophysical properties of the human ortholog of polycystin-2 under heterologous expression. To test these effects in murine primary collecting duct cells under native expression levels, we crossed the *Pkd2^del-Z^* mice with our cilia-specific reporter *ARL13B-EGFP* strain, in which all primary cilia become fluorescent under GFP excitation (DeCaen et al., 2013; Liu et al., 2018). We then isolated collecting duct cells (pIMCD) from *Pkd2^+/+^*, *Pkd2^+/del-Z^* and *Pkd2^del-Z/del-Z^* mice, and compared the CDM of polycystin-2 using the inside-out patch-clamp configuration. As expected, the voltage dependence of single channel opening for all three genotypes proportionally shifted to negative membrane potentials when internal Ca^2+^ was elevated, demonstrating that the CDM mechanism is still functional – a finding shared by our human channel results (Fig. S3). The half-maximal voltage-dependence of channel opening (*V*_1/2_) was not different from the three genotypes at low Ca^2+^. Under elevated intraciliary Ca^2+^, we observed a small but insignificant difference (−4 mV) in *V*_1/2_ when comparing the cilia channels measured from *Pkd2^+/+^* and *Pkd2^del-Z/del-Z^* mice (Table S2). The potency of Ca^2+^ opening polycystin-2 channels was not different from *Pkd2^+/+^* (EC_50_=1.2±0.4 µM), *Pkd2^+/del-Z^* (EC_50_=1.4±0.4 µM) and *Pkd2^del-Z/del-Z^* (EC_50_=1.4±0.3 µM) mice (mean±s.d.; Fig. S4, Table S2). In addition, we observed no difference in the onset of CDD between cilia currents recorded from *Pkd2^+/+^* and *Pkd2^del-Z/del-Z^* mice (Fig. S2B,D). As discussed previously, polycystin-2 was reported to function as an intracellular Ca^2+^ release channel (Koulen et al., 2002). Here, vasopressin-stimulated Ca^2+^ release was attenuated by heterologous overexpression of a truncating mutation (L703X) in a porcine kidney-derived cell line (LLC-PK1). As L703X removes the EF hand along with the entire CTD in polycystin-2, it is conceivable that removing the EF hand motif was responsible for the attenuated Ca^2+^ response to vasopressin (Koulen et al., 2002). To determine whether Ca^2+^-EF hand occupancy might affect the function of the ER-localized population of polycystin-2, we compared Gα_q_-mediated Ca^2+^ store release from primary collecting duct cells isolated from *Pkd2^+/+^* and *Pkd2^del-Z/del-Z^* mice (Fig. 6A-C). However, vasopressin- and carbachol-stimulated Ca^2+^ store release was indistinguishable between these genotypes, despite removing the affinity of Ca^2+^ for the EF hand using the del-Z mutation. Taken together, our results demonstrate that murine polycystin-2, in the ER and primary cilia membranes, is nominally impacted by the disruption of Ca^2+^-EF hand affinity, and this does not engender loss of function *in vivo* as it pertains to ADPKD.

## DISCUSSION

Many ion channels are regulated by intracellular Ca^2+^ – a feature which is tied to their physiological regulation in cell membranes. Like polycystin-2, the conductive properties of voltage-gated sodium (Na_v_s) and Ca^2+^ channels (Ca_v_s), and Ca^2+^-activated potassium channels (KCa) are modulated by internal Ca^2+^ (Xia et al., 2002; Chagot et al., 2009; Guo et al., 2016; Nejatbakhsh and Feng, 2011). Interestingly, all members of these channel families have C-terminal EF hands. However, there are clear differences in the involvement of this motif in their regulation by intracellular Ca^2+^. An example of a channel regulated by EF hand-Ca^2+^ association is the cardiac sodium channel, Na_v_1.5 (Wingo et al., 2004). Here, several missense variants associated with arrhythmia syndromes localize to the EF hand, which causes structural misfolding of the CTD and shifts Na_v_1.5 steady-state inactivation (Gardill et al., 2018). However, the EF hand is not solely responsible for Ca^2+^ regulation in these channels. Rather, the structural components of the Na_v_-Ca^2+^-sensing apparatus also include an ‘inactivation gate’ formed by their inter-domain loop motif and their CTD association with calmodulin – a ubiquitous, EF hand-containing Ca^2+^ sensor protein (Johnson et al., 2018; Shah et al., 2006; West et al., 1992). Conversely, inactivation of P/Q and L-type Ca_v_s are structurally conferred by their CTDs, but mutations which disrupt EF hand-Ca^2+^ affinity do not alter this kinetic (Zhou et al., 1997), leading researchers to explore other potential sources of the Ca^2+^-sensing mechanism (DeMaria et al., 2001; Lee et al., 1999). Based on our findings, Ca^2+^ regulation of polycystin-2 appears to be an example of the latter, as our results clearly demonstrate that Ca^2+^-EF hand association is neither required for CDM or CDD, nor is it required for *in vivo* channel function as it pertains to ADPKD. How then does polycystin-2 sense Ca^2+^ and, in response, alter its gating? We hypothesize that Ca^2+^ is acting on an undetermined receptor site(s) within the channel, or that an unknown Ca^2+^-binding protein associates with polycystin-2 to regulate its gating. Results from calorimetry and NMR spectra methods suggest that the EF hand is the only Ca^2+^ binding site in the CTD of polycystin-2 (Yang et al., 2015). However, it is important to note that the complete channel protein was not tested in these studies and in our data set, which leaves the possibility that the remaining portion of the channel may contain additional Ca^2+^ coordinating sites. As the CDM can be observed in the inside-out patch configuration, our results suggest that Ca^2+^ is either acting directly, or involves an interacting ‘Ca^2+^-sensor’ protein that is not readily disassociated from the channel, such as a pre-associated effector protein or membrane embedded factor. This feature is observed in Na_v_, Ca_v_ and in the small (SK) and large-conductance Ca^2+^-activated K^+^ (BK) channels, in which separate Ca^2+^-sensor proteins bind to motifs within the channel to control gating (Ben-Johny and Yue, 2014; Fanger et al., 1999; Lee and MacKinnon, 2018; Wang et al., 2014). Polycystin-2 interacts with a number of different proteins; however, it is unknown whether their association is Ca^2+^ dependent (Morick et al., 2013; Sammels et al., 2010; Streets et al., 2010). Recently, calmodulin was reported to bind and regulate the function of polycystin-2-like 1 – but this relationship is undetermined for polycystin-2 regulation by any other Ca^2+^ effector proteins (Park et al., 2019). It is not clear whether there is one or perhaps multiple sites that regulate CDM and CDD in either polycystin. What is clear, is that Ca^2+^ affinity for the EF hand is dispensable for the function of polycystin-2 and polycystin-2-like 1 channels (DeCaen et al., 2016). Furthermore, selectively neutralizing the EF hand vertices responsible for Ca^2+^ affinity does not result in cystic kidney disease in mice. CDM and CDD are critical forms of channel regulation, which respectively turn ‘on’ and ‘off’ the flow of Ca^2+^ within the cilia and from the ER. Thus, future work elucidating the components and structural elements responsible will be critical for understanding polycystin-2 molecular regulation, which is demonstrably involved in ciliary function and the initiation of cystogenesis in ADPKD.

We have reported that the EF hand of polycystin-2 coordinates Ca^2+^ with low affinity (*K*_d_=19 µM), in agreement with previous calorimetry studies (Ćelić et al., 2008; Yang and Ehrlich, 2016). This relatively weak affinity would seem irrelevant to ion channels of the plasma membrane, in which free Ca^2+^ (∼90 nM) is regulated by cytoplasmic buffering proteins. However, as the primary cilia retains a high concentration of Ca^2+^ (390-580 nM) at rest, and the EF hand motifs are near the ion-conducting pore, they are likely to experience elevated Ca^2+^ in the micromolar range (Delling et al., 2013). The CTD contains coiled-coil and EF hand motifs, which have structurally solved separately as fragments using crystallographic and NMR methods (Allen et al., 2014; Petri et al., 2010; Zhu et al., 2011). Previous work measuring Ca^2+^ release from ER-localized polycystin-2 has determined that truncating mutations, which also remove the EF hand, cause a complete loss of channel gating, possibly due to loss of Ca^2+^-channel affinity or lack of channel subunit oligomerization (Yang and Ehrlich, 2016; Yang et al., 2015). Although our work demonstrates that the abolishment of Ca^2+^-EF hand occupancy of polycystin-2 does not alter Ca^2+^-dependent regulation, the CTD in its entirety may still be involved in allosterically regulating the channel pore or its assembly. Based on the low resolution density maps used to solve the core structures of polycystin-2, the N- and C-terminal ends of the polycystin peptide form a multimeric structure on the internal side of the channel (Grieben et al., 2017; Shen et al., 2016; Wilkes et al., 2017). Although the contiguous CTD is not resolved either in the polycystin-2 homomeric channel structure or in its complex with polycystin-1 (Su et al., 2018), this site might be involved in controlling the opening of the lower gate through its interactions with the extended intracellular portion of the S6 helix. Our *in vivo* results demonstrate that two independent mouse models that harbor unique EF hand mutation types that abolish Ca^2+^ affinity do not manifest the polycystic kidney disease phenotype. Among ∼6000 ADPKD-associated sequences analyzed, there are no reported in-frame variants (missense, insertion and deletions) in the coding region of the *PKD2* EF hand (https://pkdb.mayo.edu/; P.C.H., unpublished data). However, there are several reported frameshift variants, which result in premature truncations of the CTD (Fig. 1B). These clinical observations, in conjunction with our findings, suggest that mutations that remove the CTD will have greater impacts on polycystin-2 gating and result in PKD-type cystogenesis, whereas those that alter Ca^2+^ occupancy of the EF hand are likely benign.

## MATERIALS AND METHODS

### CTD protein expression, purification and isothermal titration calorimetry

The C-terminal fragment of human polycystin-2 (I704-P797) was cloned into the PET19b vector and transformed into BL21 (DE3) competent cells (New England Biolabs) for bacterial expression. The translated peptide has a 10× His tag on its N-terminus. Additionally, the alanine mutants of the individual Ca^2+^-coordinating residues were created and transformed. Cells were grown in 2× YT broth in a 37°C shaking incubator until OD_600_ reached ∼0.6, and were induced by 0.4 mM isopropyl 1-thio-β-D-galactopyranoside and grown for another 4 h. Cells were harvested and resuspended in buffer A (500 mM NaCl, 20 mM Tris-HCl, pH 7.4), 5% glycerol, 0.25 mM EDTA (pH 8.0), 1 mM PMSF (RPI), 25 µg/ml lysozyme and DNase I (GoldBio). The suspension was then lysed by sonication and clarified by centrifugation (30,000 ***g*** for 0.5 h). Filtered supernatant was loaded into a HisPur cobalt Superflow agarose column (Thermo Scientific). The column was washed with 10 column volumes (CV) of buffer A, 5 CV of buffer A plus 5 mM imidazole, and finally eluted with 3 CV of buffer A+100 mM imidazole. The eluate was desalted by dialysis in 150 mM NaCl, 20 mM Tris/HCl, 20 mM imidazole (pH 7.4). SDS-PAGE confirmed the expression, solubility and purity of the peptide. All of our solutions were formulated with ultrapure water (Milli-Q IQ 7005 water purification system), which has less than 0.29 ng/l Ca^2+^ ions present. The binding affinity was recorded based on the heat difference between the sample cell and reference cell measured by MicroCal iTC200. Forty successive additions of 1 µl CaCl_2_ (2 mM) were added into the sample cell containing 400 µM of the respective purified peptides at 1 min intervals. The cells were insulated by an adiabatic jacket held at 25°C. The heat of dilution of Ca^2+^ was not subtracted from data sets as the signal was prohibitively small for several of the mutant channels. The exothermic energy of the bimolecular interaction between Ca^2+^ and peptide during each injection was analyzed by integrating the change in heat to generate the binding isotherm. The resulting relationship was fit (Origin 7.0) with a one-site independent binding model to determine the binding affinity (*K*_d_).

### Production of the *Pkd2^-X-Z^* mouse strains and histology analysis

Signal guide RNAs (sgRNAs) for the T769A and E772A mutations (5′-GCTCACGCTCGGTCAGTTCC-3′) and the inserted V5 tag (5′-CACGTGTGGATTATTAGGCA-3′) were designed using the CRISPR Design tool (http://crispr.mit.edu/). To edit the mouse genome, single-stranded oligodeoxynucleotide (ssODN) donor templates for T769A and E772A point mutations, and another with a V5 tag in-frame at the C-terminus, were synthesized (IDT). A sgRNA plasmid was constructed and linearized, followed by *in vitro* transcription of sgRNAs using MEGAshortscript Kit (Invitrogen). The yield and quality of sgRNA were assessed by absorbance ratio and gel electrophoresis (Shen et al., 2014). *In vitro* transcribed sgRNAs, Cas9 protein (NEB) and two donor ssODN templates were microinjected into zygotes, followed by culture and transfer of blastocysts into the uterus of pseudopregnant ICR female mice (Horii et al., 2014; Wang et al., 2013). Founders were identified by PCR amplification of genomic DNA from tail biopsies, followed by sequencing of the PCR products (Shen et al., 2014). Genotyping of experimental mice was carried out using allele-specific PCR primers (capitalized) for the T769A and E772A double mutant (TCAAGAGCTTGCTGAACGAGC and tgtttaccaaggtcttgggcaagca) and wild-type alleles (CCAGGAACTGACCGAGCGTGA and tgtttaccaaggtcttgggcaagca).

### Production of the *Pkd2^del-Z^* mouse strains, MRI and kidney histology analysis

The *Pkd2^del-Z^* mouse was generated using the CRISPR/Cas9 method with the guide RNA 5′-AAACAGCGTGAGCATCAACAGATGC-3′. To characterize their phenotype *Pkd2^+/+^* (10 males and 8 females), *Pkd2^+/del-Z^* (10 males and 9 females) and *Pkd2^del-Z/del-Z^* (10 males and 8 females) were MRI scanned at 9 and 12 months of age. Nine-month-old *cPkd2* mice (10 males and 8 females) were fed doxycycline through drinking water for 1 week to genetically attenuate *Pkd2* expression (Pax8^rtTA^; TetO-cre; Pkd2^fl/fl^), as described previously (Liu et al., 2018; Ma et al., 2013). MRI imaging was conducted at the Northwestern University Center for Advanced Molecular Imaging using a Bruker BioSpec (9.4 Tesla). Mice were anesthetized and placed in a chamber containing 3% isoflurane, and their respiration was monitored for the duration of the scan (Irazabal et al., 2015). The kidneys from *Pkd2^+/+^*, *Pkd2^+/del-Z^*, *Pkd2^del-Z/del-Z^* and *cPkd2* mice (10 mice each) were fixed in 40% paraformaldehyde for 24 h, sectioned on a cryostat, mounted on glass coverslips and stained with hematoxylin and eosin (H&E). Images of medial sections from both kidneys were analyzed using FIJI (ImageJ) to identify cystoid foramen. Images were processed as black and white, and reverse-negative. The images were then analyzed using the particle analysis search protocol, in which the lower limit threshold for circular foramen was set to 20 µm to identify cystoid foramen in the tissue samples. *Pkd2^del-Z^* mice were crossed with our previously established *ARL13B-EGFP* mouse, so that primary cilia could be visualized from living cells during our cilia electrophysiology recordings described in the next section (Liu et al., 2018). Animals were housed at AALAS certified facilities located at Yale University, Northwestern University and the Mayo Clinic. All animal procedures and protocols were approved by the Institutional Animal Care and Use Committees (IACUCs) of each university.

### Cell culture of primary inner medullary collecting duct cells and immortalized cell lines

Primary inner medullary collecting ducts cells (pIMCD) were isolated from wild-type or *Pkd2^del-z/del-z^* mice co-expressing the *ARL13B-EGFP* cilia reporter, using a method described previously (Liu et al., 2018). Briefly, inner medullae were removed from the kidney and disassociated using a Dulbecco's PBS containing 2 mg/ml collagenase A and 1 mg/ml hyaluronidase. After mechanical disassociation on ice, medullary cells were cultured in Dulbecco's modified essential medium (DMEM) supplemented with 10% fetal bovine serum (FBS) and 100 units/ml penicillin and 100 μg/ml streptomycin. Cilia were patched from cells within 6 days after isolation, and within one passage. HEK *PKD2*^Null^ cell lines were generated using the CRISPR/Cas9 gene editing kit available from Addgene and authenticated using PCR analysis, as described previously (Liu et al., 2018). To generate the stable cell lines expressing C-terminally tagged versions of wild-type and deletion mutants of human polycystin-2, the human (h)PKD2 gene was subcloned into the lentiviral pLVX-GFP-N1 (Clontech) vector using the Gibson assembly method. The del-Z deletion in hPKD2-GFP was generated using a modified site-directed mutagenesis protocol. Lentiviral infected cells were selected using culture medium containing puromycin (2 μg/ml), and sorted (BD FacsMelody) at 5000 to 10,000 counts per minute to enrich for the transgene expression. Stable cell lines were cultured in DMEM supplemented with 10% FBS and 100 units/ml penicillin, 100 units/ml streptomycin and 1 μg/ml puromycin selection antibiotic. On a monthly basis, mycoplasma testing (MycoProbe, R&D systems) was performed on all active cultures in our incubators.

### Electrophysiology

The electrophysiologist was blinded by a third party in the laboratory, whereby test groups were assigned a letter to conceal the genetic identity of the cells being evaluated. The identity of the cells remained unknown by the electrophysiologist until the analysis was complete. Ciliary ion currents were recorded using borosilicate glass electrodes polished to resistances of 14-23 MΩ using the cilium patch method described previously (Vien et al., 2020). Single channel currents measured in the inside-out configuration were recorded in symmetrical sodium concentrations. All of our solutions were formulated with ultrapure water (Milli-Q IQ 7005 water), which has less than 0.29 ng/l Ca^2+^ ions present. The internal solution (bath) contained (in mM): 120 NaMES, 10 NaCl and 10 HEPES. Ca^2+^ was buffered with 5 EGTA, 5 Na_4_-BAPTA {Glycine, N,N′-[1,2-ethanediylbis(oxy-2,1-phenylene)]bis[N-(carboxymethyl)]-,tetrasodium} and 0.5 EDTA; free Ca^2+^ was calculated using Maxchelator and titration of 1 M CaCl_2_ solution (Bers et al., 2010); pH was adjusted to 7.3 using NaOH. Standard external solution (pipette electrode) contained 150 NaCl, 10 HEPES, 2 CaCl_2_; pH 7.4. All solutions were osmotically balanced to 300 (±7) mOsm with d-mannitol. Whole-cell currents used to measure CDD were also recorded in symmetrical sodium concentration, placing the internal recording solution in the pipette electrode and the external recording solution in the bath. Data were collected using an Axopatch 200B patch-clamp amplifier, Digidata 1550B and pClamp 10 software. Single channel currents were digitized at 50 kHz and low-pass filtered at 10 kHz. Intraciliary conditions were controlled using an Octaflow II rapid perfusion system (ALA systems) in which the patched cilia and electrode were held in the perfusate stream. Data were analyzed by Igor Pro 7.00 (Wavemetrics). The polycystin-2 open probability (Po) current-voltage relationships were fitted to a Boltzman equation, f(x)=1/(1+exp[*V*-*V*_1/2_]/k), to estimate the half-maximal voltage (*V*_1/2_) required to open the channels. The potency of Ca^2+^ opening polycystin-2 channels was estimated by integrating the single channel current measured in response to elevating the internal Ca^2+^ concentration ([Ca_in_]). The average integrated current was fitted to the Hill equation, f(x)=base+(max-base)/{1+(EC_50_/[Ca_in_]), to estimate the effective concentration of Ca^2+^ (EC_50_) required to half maximally stimulate the polycystin-2 response.

### Intracellular Ca^2+^ measurements using Fura-2

Vasopressin-mediated intracellular Ca^2+^ responses were measured from pIMCD cells 24 to 72 h after cells were isolated. Before seeding the cells, glass-bottom dishes were pre-coated with poly-L-lysine and laminin (Sigma-Aldrich). The cells were incubated for 1 h at 37°C in complete medium after loading with 2 µg of Fura-2/AM (Invitrogen). The cells were then washed with and stored for 15 min in Tyrode's solution (in mM): 140 NaCl, 4 KCl, 2 MgCl_2_, 2 CaCl_2_, 10 Glucose and 10 HEPES. Cells were adjusted in pH to 7.4 with NaOH and osmolarity to 300 mOsm with d-mannitol. Cells were then placed under an inverted wide-field microscope equipped with a 20× objective lens (Olympus IX81), and the stage temperature was held at 37°C (Tokai Hit). Images of Fura-2 fluorescence remission at 520 nm were captured every 2 s during excitation at 380 nm (Ca^2+^ free) and 340 nm (Ca^2+^ bound). Images were acquired and analyzed using SlideBook (Intelligent Imaging Solutions), which synchronizes the filter wheel changer (Lambda 10-3, Sutter Instrument) and the camera (ImagEMX2, Hamamatsu). In each trial or replicate (*n*), the emission fluorescence (340:380 nm) ratio of 15 to 30 cells was recorded after subtracting the background fluorescence levels. This ratio was averaged at the start (for resting level) and the response was reported after extracellular vasopressin or carbachol treatment (for maximal response). At least three replicates (*n*) were performed with cells isolated from five animals of the same genotype.

### Statistical analysis

Sample sizes were determined based on 2-sample *t*-test power analysis of the recorded variance from pilot study results from each assay, where target power=0.9 and α=0.05. Statistical comparisons were made using one-way ANOVA or two-tailed unpaired Student's *t*-tests using OriginPro software (OriginLab) or Excel (Microsoft). Experimental values are reported as the mean±s.d., unless otherwise stated. Differences in mean values were considered significant at *P*<0.05. All of our results are normally distributed as per a Shapiro–Wilk Test.

## Supplementary Material

Supplementary information
