# Supplementary material for: Disrupting polycystin-2 EF hand Ca2+ affinity does not alter channel function or contribute to polycystic kidney disease
Source: J Cell Sci. 2020 Dec 24;133(24):jcs255562. doi: 10.1242/jcs.255562 (PMC7774883; doi:10.1242/jcs.255562)
Supplement: Supplementary information [file joces-133-255562-s1.pdf]

Channel	Kd, EF hand affinity for Ca^{2+}, (±S.D)
Human PKD2, WT	19 μM ± 5
X (D763A)	1.3 mM ± 0.2
Y (D765A)	1.1 mM ± 0.1
Z (D767A)	890 μM ± 32
-X (T771A)	129 μM ± 15
-Z (E774A)	298 μM ± 25
-X-Z (T771A:E774A)	*Not detected* (> 2 mM)
del-Z (H773_E774del)	*Not detected* (> 2 mM)

Table S1. Isothermal calorimetry results measuring Ca^{2+}-EF hand affinity (Kd) of Polycystin-2. Tabulated average and standard deviation of calcium affinity results derived from the calorimetry experiments described in Figure 1 and Supplementary Figure 1.

Polycystin-2 orthologue or mutation	Ca^{2+} potency, EC_{50}; Hill coefficient	$V_{1/2}$ $[Ca^{2+}]_{in}$ = 100 nM	$V_{1/2}$ $[Ca^{2+}]_{in}$ = 100 µM	$\Delta V_{1/2}$
Human *PKD2*, WT	0.92 µM ± 0.4; 0.95 ± 0.3	78 mV ± 8	31 mV ± 7	-47 mV
Human *PKD2*, -X-Z	0.98 µM + 0.3 (*P* = 0.7); 0.96 ± 0.2	76 mV ± 6 (*P* = 0.3)	30 mV ± 8 (*P* = 0.9)	-46 mV
Mouse *Pkd2*$^{+/+}$	1.2 µM ± 0.4; 0.94 ± 0.3	79 mV ± 8	32 mV ± 7	-47 mV
Mouse *Pkd2*$^{+/del\text{-}Z}$	1.4 µM ± 0.4 (*P* = 0.4); 0.95 ± 0.3	82 mV ± 8 (*P* = 0.7)	34 mV ± 8 (*P* = 0.6)	-48 mV
Mouse *Pkd2*$^{del\text{-}Z/del\text{-}Z}$	1.4 µM ± 0.3 (*P* = 0.4); 0.94 ± 0.3	83 mV ± 7 (*P* = 0.3)	36 mV ± 6 (*P* = 0.4)	-47 mV

Table S2. Cilia electrophysiology results measuring Ca^{2+} potency for polycystin-2 activation (EC_{50}) and CDM of voltage dependent gating ($V_{1/2}$). Tabulated average EC_{50} and $V_{1/2}$ values derived from Figure 2, 3 and Supplemental Figures 3, 4.

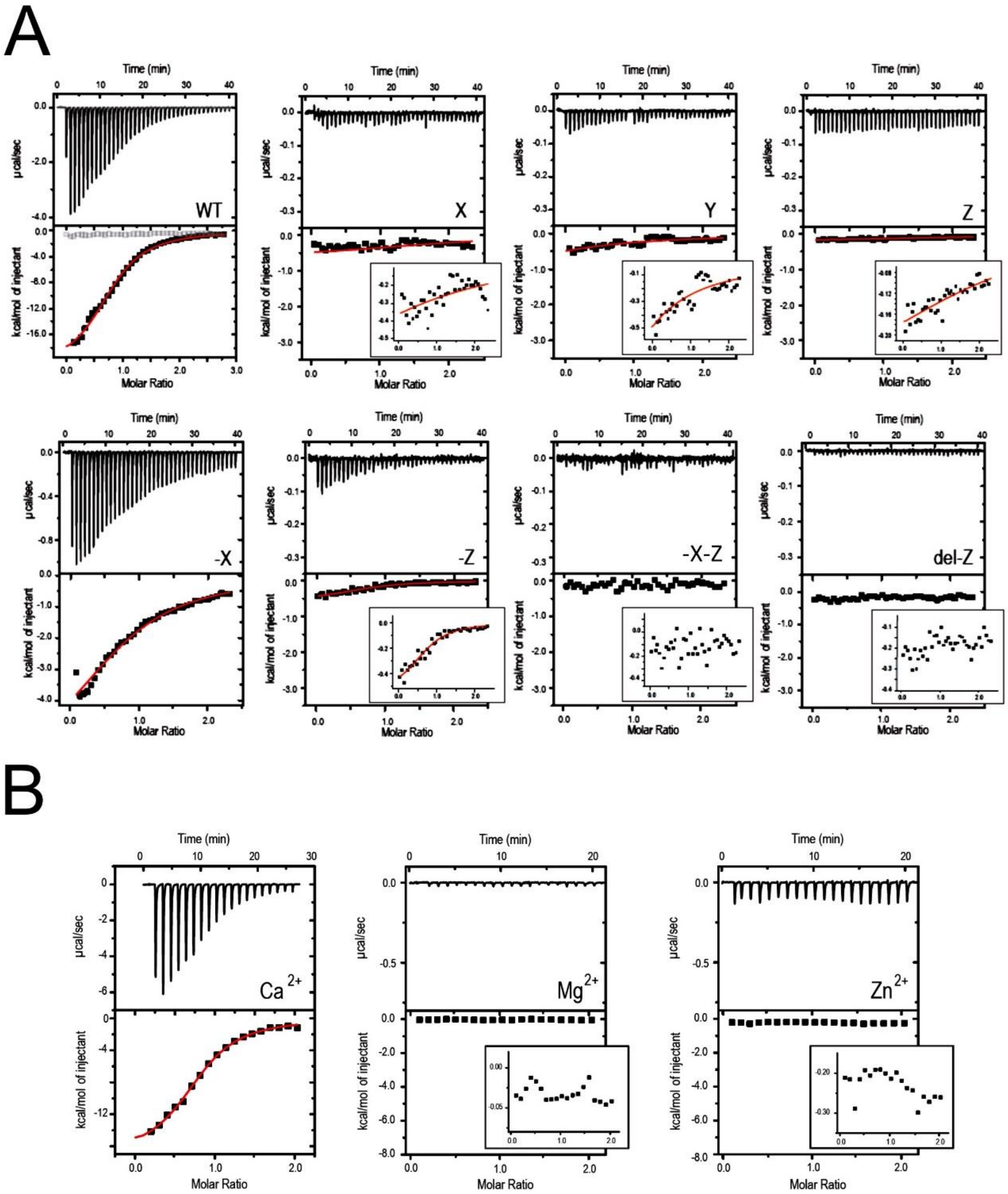

Figure S1. Calorimetric titration of divalent ions into the CTD of polycystin-2.

A) ITC profiles corresponding to calcium binding to the wild type or mutant polycystin CTD (704-797). The upper panel of each profile shows the raw data of the heat changes upon successive injection of 1 µL $CaCl_2$, performed at 25°C. The bottom panel shows the binding curve where the peaks of the heat change were integrated and plotted against the molar ratio of accumulated calcium to the concentration of the protein fragment. The line represents a non-linear fit based on a single-site binding model. The stoichiometry of Ca^{2+} to WT CTD peptide (N) = 0.75 ± 0.06: the change in enthalpy (ΔH) = -17.7 ± 1.5 KJ/mol and entropy (ΔS) - 40.3 ± 5.3 kcal/mol•K; Error = SEM. **B**) Exemplar profiles demonstrate the lack of Zn^{2+} and Mg^{2+} affinity for the polycystin-2 CTD. Note the difference in time scales between the data sets shown in A and B. In some cases, the binding curves were expanded in the inset for better visualization

Journal of Cell Science • Supplementary information

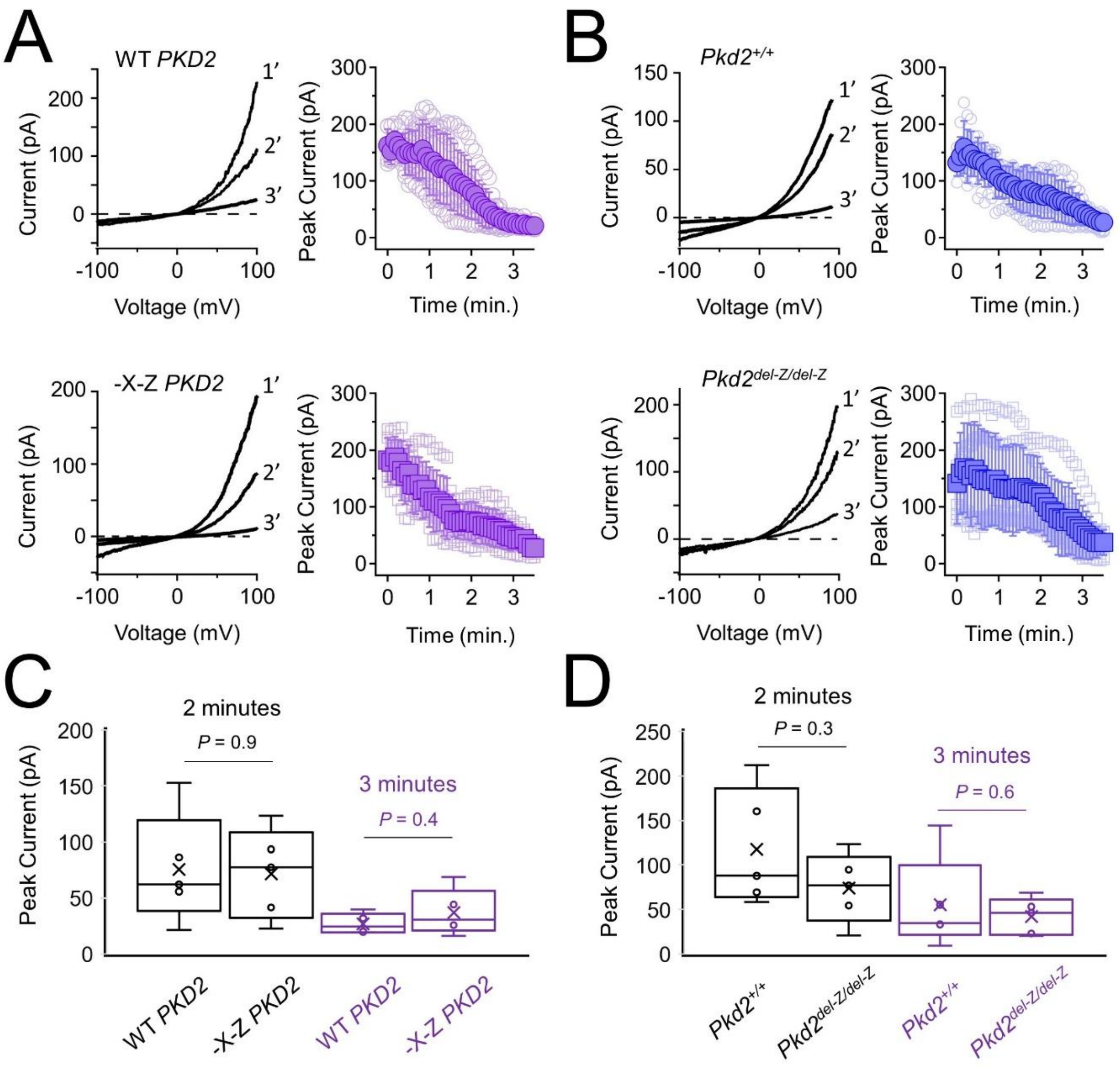

Figure S2. Ca^{2+}-dependent desensitization (CDD) of human and mouse polycystin-2 channels. A) *Left*, desensitization of whole cilia currents measured from HEK cells expressing WT or -X-Z EF hand *PKD2* mutant channels under high intracellular free calcium (30 μM) captured at minute time intervals (1’, 2’ and 3’). *Right*, corresponding time courses of desensitization plotted from individual cilia patches (open symbols) and the average responses (filled symbols). **B**) Same as in A, but measuring CDD from pIMCD cells isolated from *Pkd2*$^{+/+}$ or *Pkd2*$^{del\text{-}Z/del\text{-}Z}$ mice. **C, D**) Box (S.E.M.) and whisker (S.D.) plots comparing peak current magnitudes measured from A and B at two and three minute intervals. Error is equal to S.D., N =7 cilia for each genotype. *P* values resulting from Student’s t test analysis are shown above the graphs.

Journal of Cell Science • Supplementary information

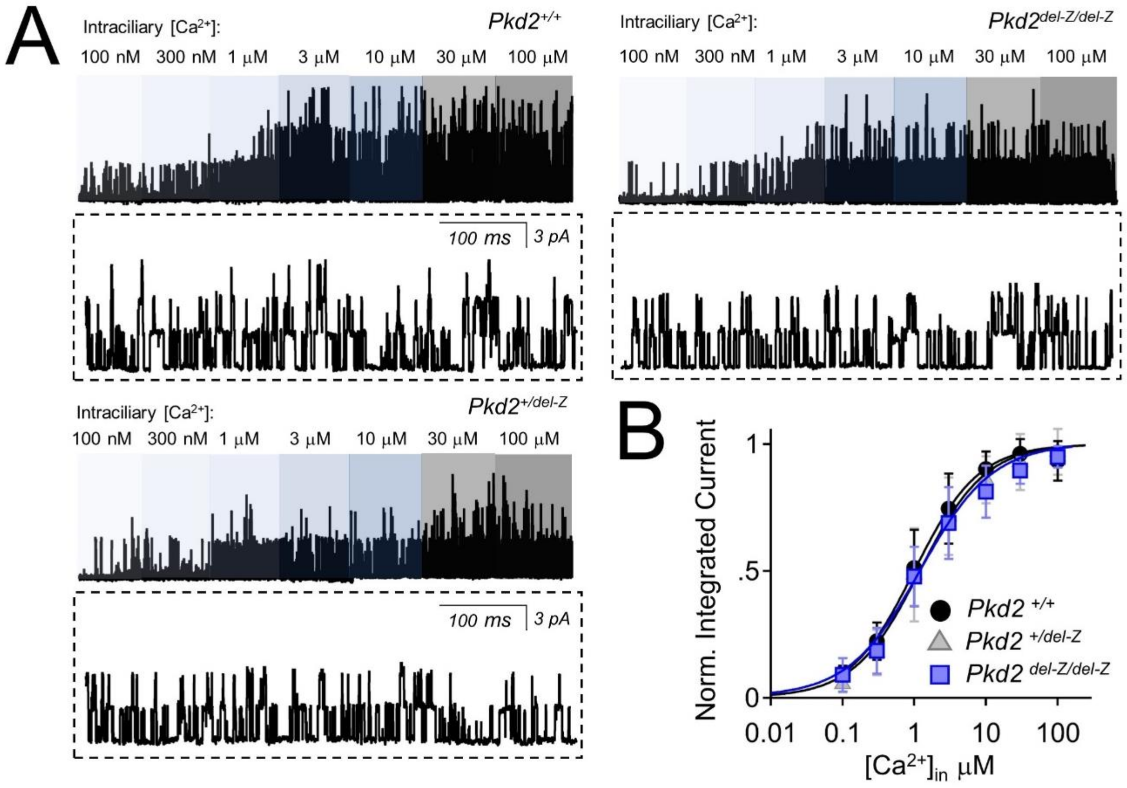

Figure S3. Ca^{2+} equally activates primary cilia currents from mice expressing WT and EF hand mutant *Pkd2$^{del\text{-}Z}$* alleles. **A**) Polycystin-2 single channels recorded from primary cilia of collecting duct cells isolated from mice expressing WT and *Pkd2$^{del\text{-}Z}$* alleles. Using the same protocol described in figure 2, polycystin-2 channel open events were captured in the inside-out configuration and intraciliary calcium was raised in the perfusate. The traces within the dashed square show the expanded time scale in the 100 µM calcium condition. **B**) The relationship of ciliary calcium and normalized integrated current from genotypes (N = 7 cilia, Error = S.D.).

Journal of Cell Science • Supplementary information

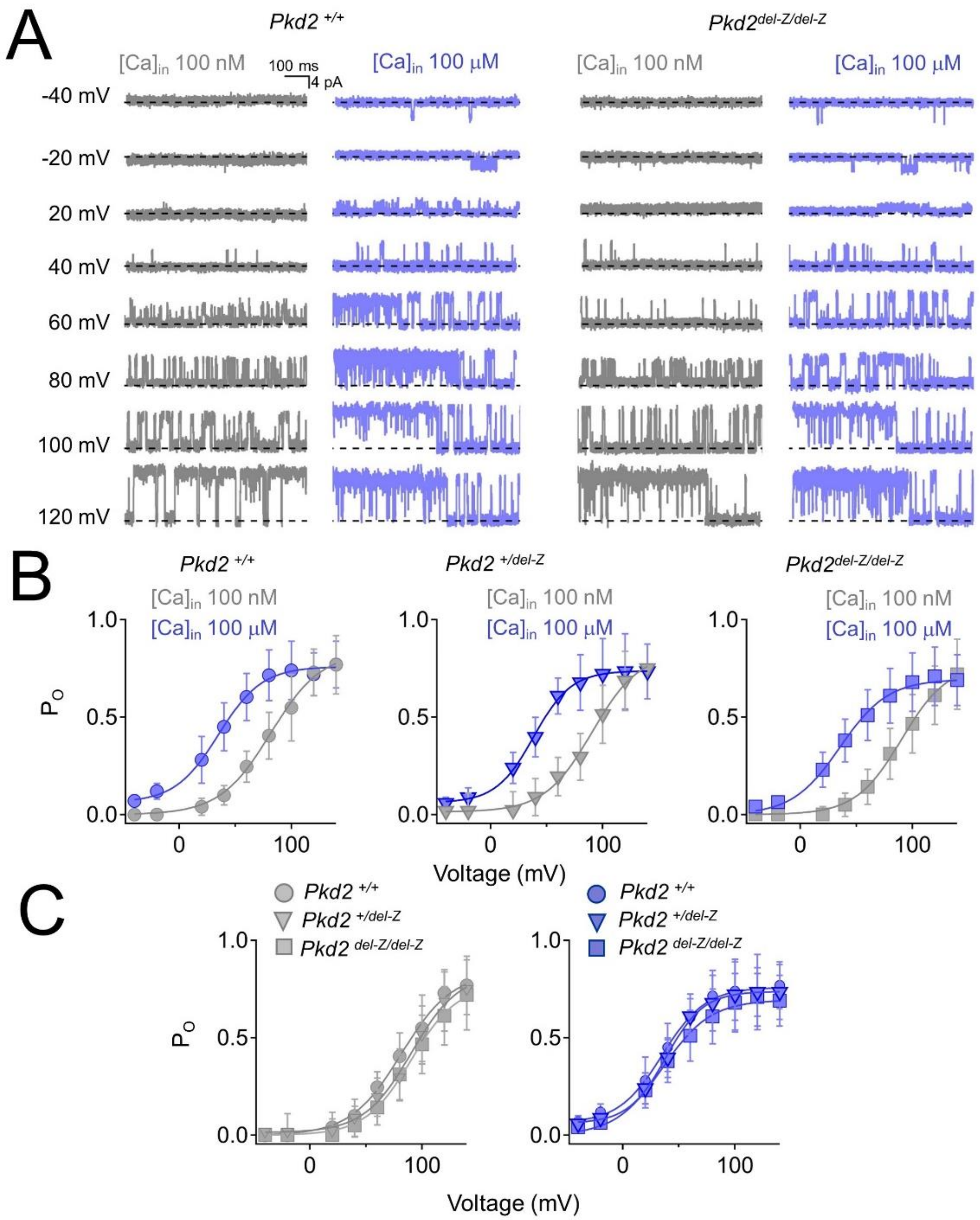

Figure S4. CDM of ciliary polycystin-2 is similar from mice expressing WT and *Pkd2*$^{del\text{-}Z}$ alleles. **A)** *E*xemplar polycystin-2 single channel recordings measured in the inside-out cilia patch configuration. pIMCD cells were isolated from the kidney medulla of mice expressing WT and del-Z alleles that co-expressed the *ARL13B-GFP* transgene. **B, C**) Average channel open probability plotted as a function of voltage in the presence of 100 nM and 100 µM intracellular calcium. The data was fit the Boltzmann equation to estimate the half-maximal voltage response ($V_{1/2}$). Error bars indicate S.D. from 6 cilia recordings from each cell type.

Journal of Cell Science • Supplementary information

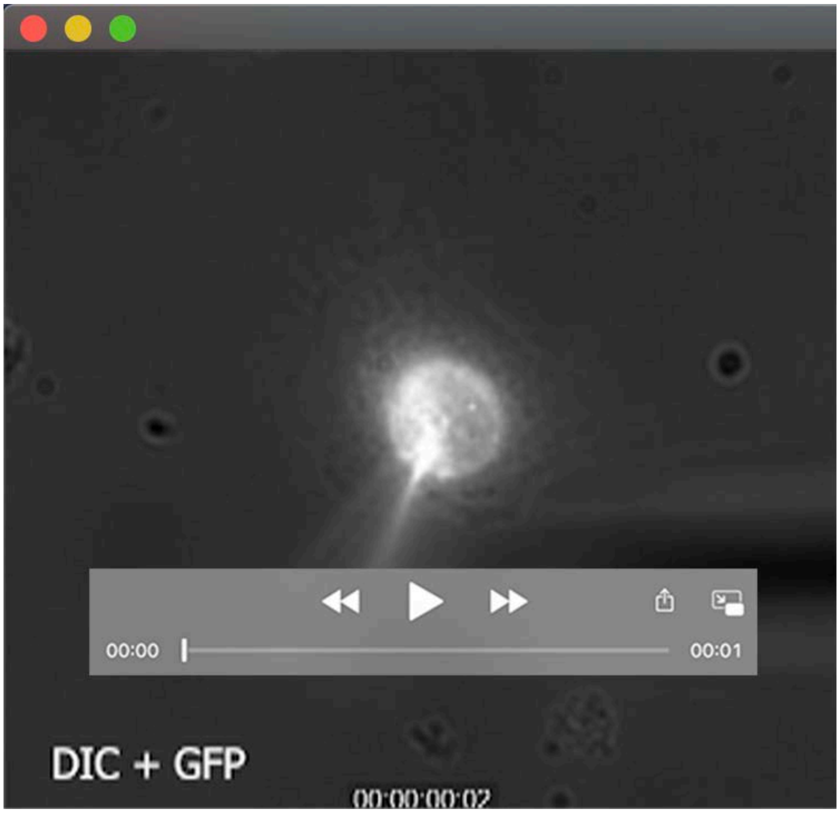

Movie 1. Visualization of the GFP-illuminated primary cilia membrane used to establish high-resistance seals necessary to record polycystin-2 channel current.
